# Cuproptosis: unveiling a new frontier in cancer biology and therapeutics

**DOI:** 10.1186/s12964-024-01625-7

**Published:** 2024-05-01

**Authors:** Ying Feng, Zhibo Yang, Jianpeng Wang, Hai Zhao

**Affiliations:** 1 Department of Emergency, the Affiliated Hospital of Qingdao University, No. 16 Jiangsu Road, Qingdao, 266005 Shandong China; 2 Department of Neurosurgery, 3201 Hospital of Xi’an Jiaotong University Health Science Center, Hanzhong, 723000 Shaanxi China; 3 Department of Neurosurgery, the Affiliated Hospital of Qingdao University, No. 16 Jiangsu Road, Qingdao, 266005 Shandong China

**Keywords:** Cuproptosis, Copper, Immunotherapy, Targetted therapy, Copper homeostasis, Mitochondria

## Abstract

Copper plays vital roles in numerous cellular processes and its imbalance can lead to oxidative stress and dysfunction. Recent research has unveiled a unique form of copper-induced cell death, termed cuproptosis, which differs from known cell death mechanisms. This process involves the interaction of copper with lipoylated tricarboxylic acid cycle enzymes, causing protein aggregation and cell death. Recently, a growing number of studies have explored the link between cuproptosis and cancer development. This review comprehensively examines the systemic and cellular metabolism of copper, including tumor-related signaling pathways influenced by copper. It delves into the discovery and mechanisms of cuproptosis and its connection to various cancers. Additionally, the review suggests potential cancer treatments using copper ionophores that induce cuproptosis, in combination with small molecule drugs, for precision therapy in specific cancer types.

## Introduction

In recent years, the discovery of cuproptosis has not only challenged the conventional understanding of the role of copper in cellular death mechanisms but also opened new avenues in cancer research. From our perspective, cuproptosis represents a paradigm shift suggesting our approach to cancer therapeutics may be fundamentally transformed by targeting copper metabolism [1]. Copper, an essential trace element, plays a pivotal role in numerous cellular signaling pathways and is linked to cancer biology [2–5]. Historically, the pathways and forms of copper-induced cell death were not well-defined until a study suggested cuproptosis as a distinct mechanism, closely associated with mitochondrial respiration and the lipoic acid pathway, marking a significant advancement in understanding the role of copper in cell death [1]. A significant number of researchers are investigating the critical connection between cuproptosis and various types of cancer [6–8]. Strong association has been identified with cellular metabolism and the heightened levels of aerobic respiration seen in certain cancers like melanoma, breast cancer, and leukemia [9–12]. This relationship extends to cancers harboring cancer stem cells and those resistant to drugs, where a high mitochondrial metabolic rate is observed [13]. Studies are increasingly focusing on the expression levels of key genes involved in cuproptosis and their correlation with tumor prognosis, emphasizing the importance of understanding this link for future therapeutic strategies.

**Fig. 1:**
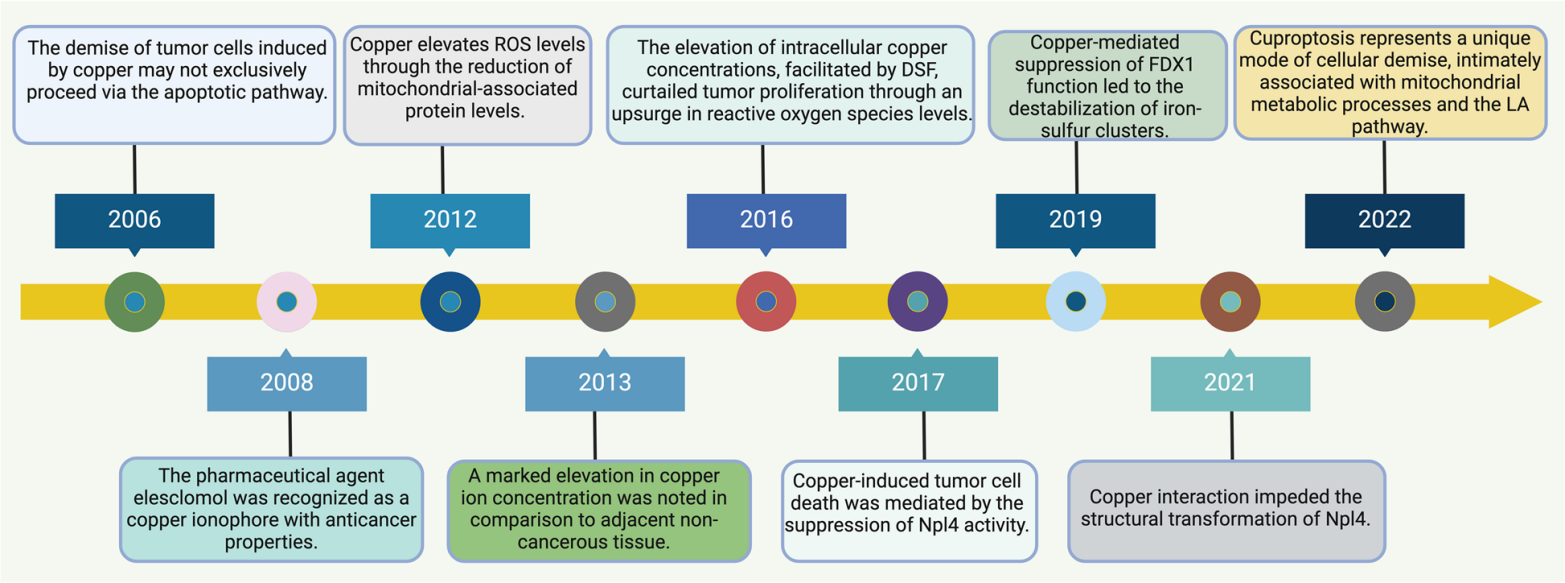
The timeline of the discovery of cuproptosis. Abbreviation: ROS, reactive oxygen species; DSF, Disulfiram; FDX1, Ferredoxin 1; LA, Lipoic Acid

This review embarks on an in-depth exploration of the dual role of copper within biological systems—essential for various cellular functions yet potentially harmful when dysregulated. We traverse the landscape of copper metabolism and homeostasis, laying the groundwork for understanding how aberrations in these processes contribute to cancer development. The elucidation of cuproptosis molecular mechanisms presents an evident contrast to traditional cell death pathways, underscoring its unique influence on cancer cell fate **(**Fig. 1**)**. Further, we dissect the implications of copper dysregulation in the oncogenic process, from tumor initiation to metastasis, and deliberate on the innovative therapeutic strategies targeting this newly discovered cell death form. By integrating insights from translational research and clinical trials, we aim to illuminate the future trajectory of cuproptosis research and its potential to redefine cancer treatment paradigms. Through a synthesis of current knowledge and prospective inquiry, this article endeavors to chart the course for future investigations, poised to unlock the full therapeutic potential of targeting cuproptosis in the fight against cancer.

### Regulation of copper metabolism and homeostasis in healthy and cancer cells

**Fig. 2:**
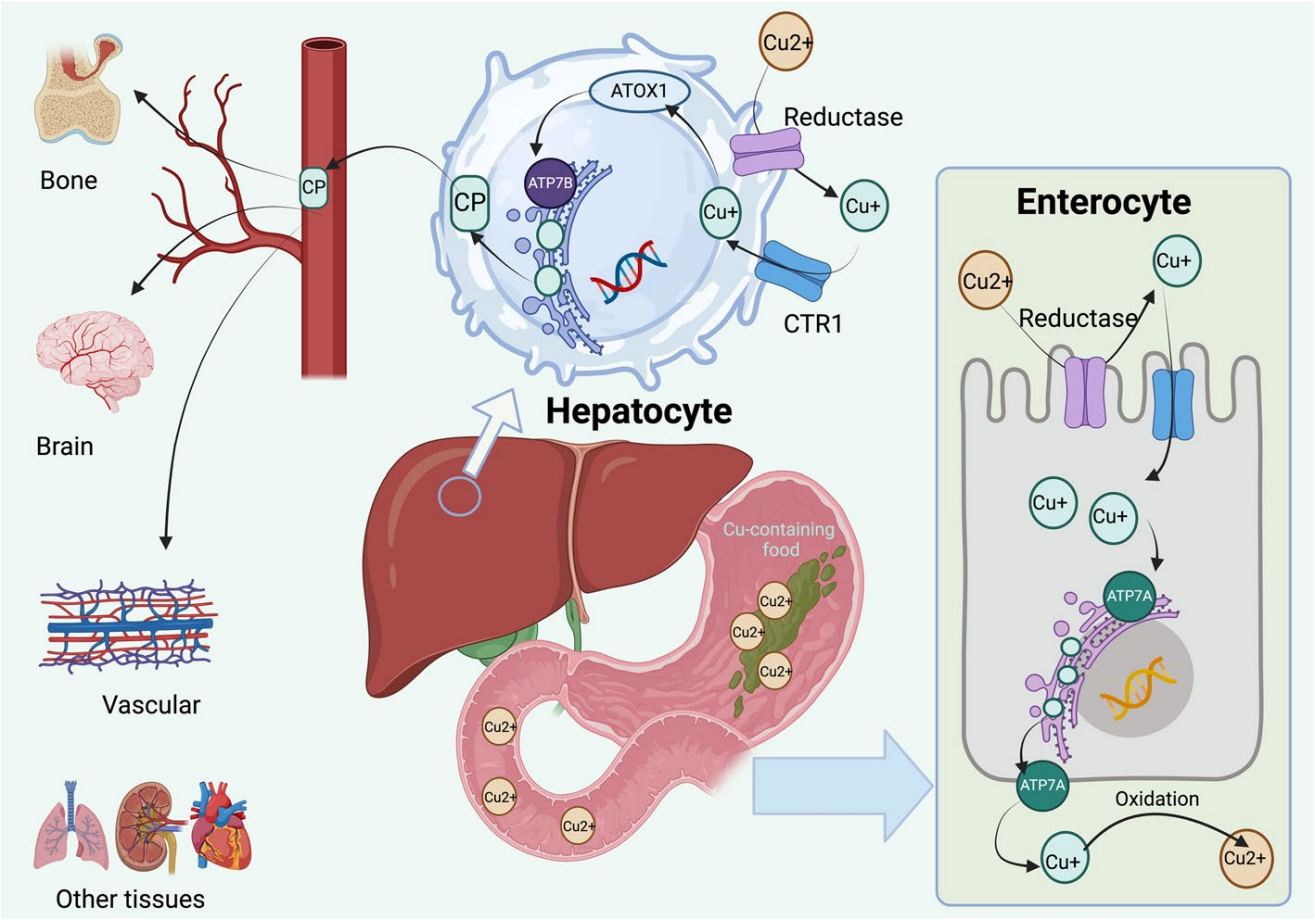
Copper metabolism and homeostasis in the human body. The intricate mechanism of copper absorption and metabolism within the human body commences with the ingestion of dietary Cu(II), which is absorbed in the stomach and small intestine. Subsequent to absorption, Cu(II) undergoes reduction to Cu(I) by a reductase enzyme. This monovalent copper is then bound by chaperone proteins, notably ATOX1, and is transported to the liver for further utilization and regulation. Within the liver, ATP7B plays an instrumental role in the integration of copper into ceruloplasmin, which serves as a carrier for copper in the bloodstream to various tissues, and in mediating the excretion of surplus copper into bile for elimination. The function of liver is crucial in preserving copper homeostasis, ensuring the prevention of both copper deficiency and toxicity. Additionally, another transporter, ATP7A, is primarily tasked with exporting copper from intestinal epithelial cells into the bloodstream, aiding in the oxidation of Cu(I) back to Cu(II), which facilitates its release and distribution throughout the body. Abbreviation: ATOX1, Antioxidant 1 Copper Chaperone

While the critical role of copper metabolism and homeostasis in safeguarding cellular integrity is well documented, the complexity of the dual role of copper — as both a vital nutrient and a potential cellular toxin—underscores the necessity for a nuanced understanding of its metabolic pathways. The intricate balance required to maintain copper homeostasis highlights potential vulnerabilities in cancer cells that could be exploited therapeutically [14–16]. Copper metabolism begins with the absorption of copper from the diet via the gastrointestinal tract, followed by its conveyance to the liver, which acts as a hub for its distribution to various bodily tissues or its integration into enzymatic systems vital for metabolic and protective cellular activities [17, 18]. Copper chaperones play a pivotal role in directing copper to its precise intracellular destinations, facilitating its incorporation into essential enzymes involved in energy metabolism and the mitigation of oxidative stresss [19, 20]. The cellular machinery, including ATPase Copper Transporting Alpha (ATP7A) and ATPase Copper Transporting Beta (ATP7B) transporters, orchestrates the regulation of copper levels by mediating its efflux from cells or directing its elimination through the bile, thus preventing toxic accumulation [21] (Fig. 2). Both ATP7A and ATP7B are pivotal copper-transporting ATPases, each playing distinct yet complementary roles in copper homeostasis across various cell types [21–23]. ATP7A, predominantly expressed in tissues like the intestine, brain, and placenta, facilitates the efflux of copper from cells, thereby aiding in the absorption of dietary copper and its delivery to peripheral tissues [24]. Conversely, ATP7B, primarily located in the liver, plays a critical role in the excretion of excess copper into the bile and its incorporation into ceruloplasmin [25]. The differential expression and localization of ATP7A and ATP7B underscore their specialized functions in maintaining copper balance within the body, preventing copper deficiency and toxicity. Their presence in diverse cell types not only highlights the systemic importance of copper regulation but also suggests potential therapeutic targets for disorders related to copper metabolism, such as Menkes and Wilson's diseases. Insights into these sophisticated regulatory mechanisms are fundamental for exploring the implications of copper dysregulation in disease pathogenesis and for the advancement of therapeutic strategies targeting such imbalances.

#### Copper uptake

In the human gastrointestinal system, the assimilation of dietary copper predominantly takes place within the small intestine [26]. It is within this environment that extracellular Cu(II) ions are present. For adults, the daily copper intake recommended is 0.9 mg [27]. The absorption process of Cu(II) at the intestinal level necessitates its prior reduction to Cu^ + facilitated by the binding to the six transmembrane epithelial antigen of the prostate (STEAP) family of metalloreductases, which consists of STEAP1 to STEAP4, all exhibiting intrinsic metal reductase activity [28]. The passage of Cu^ + through the gastrointestinal lining is chiefly mediated by the solute carrier family 31, specifically member 1 (solute carrier family 31 member 1 or copper transporter 1) and member 2 (solute carrier family 31 member 2 or copper transporter 2), with multiple mechanisms potentially influencing SLC31A1’s activity, thereby regulating copper absorption [29]. The activity of solute carrier family 31 member 1 (SLC31A1) can be modulated through the recruitment of histone demethylase jumonji domain-containing protein 2a (JHDM2A) by Zinc finger protein 711 (ZNF711) to the SLC31A1 promoter, leading to reduced levels of Histone 3 lysine 9 methylation 2 (H3K9me2) and the activation of SLC31A1 transcription [29]. Additionally, the copper-responsive element Specificity protein 1 (SP1) transcriptionally governs SLC31A1 expression, thus maintaining a balance between cellular copper and SP1 levels [30–32]. Polypyrimidine tract-binding protein 1 (PTBP1) interacts directly with SLC31A1 mRNA, reducing SLC31A1 expression by destabilizing its mRNA [33]. Under conditions of vascular endothelial growth factor (VEGF) stimulation, SLC31A1 undergoes rapid sulfenylation at cysteine 189, facilitating the formation of a disulfide bond between SLC31A1 and VEGF receptor 2 (VEGFR2), leading to their co-internalization into early endosomes, which in turn promotes sustained VEGFR2 signaling and angiogenesis [34]. Furthermore, exposure to copper and cisplatin triggers the internalization and degradation of SLC31A1 [35]. In the scenario of diminished SLC31A1, copper uptake via SLC11A2 or divalent metal transporter 1 acts as a compensatory mechanism [36]. The functions of SLC31A1 and SLC31A2 are intricately linked within the copper homeostasis framework, with SLC31A1 ensuring the stability of SLC31A2, which in turn influences the generation of a truncated form of SLC31A1 devoid of its copper-/cisplatin-binding ectodomains [36]. Notably, both transporters are associated with platinum sensitivity in certain cancers [37]. AMPK, upregulated by glucose restriction via ROS, increases copper transporter 1 (CTR1) expression, underscoring the need for comprehensive investigations into whether these membrane transporters perform cancer-specific roles in modulating copper uptake [38]. Mucin 2 in the intestine, by binding copper at two distinct sites (Cu(II) and Cu(I)), mitigates copper toxicity and conserves dietary antioxidants, thereby facilitating the cellular entry of such essential trace metals [39]. Elevated copper levels are observed in both inflamed and cancerous tissues, with inflammatory cytokines like interleukin (IL) -17 enhancing cellular copper uptake through STEAP4 induction [40]. Copper plays a significant role in the carcinogenic B-Raf proto-oncogene (BRAF) signaling pathway [41]. Intracellular copper levels, when elevated by IL-17, activate the E3 ubiquitin-protein ligase x-linked inhibitor of apoptosis (E3-ligase XIAP). This activation enhances the nuclear factor kappa-light-chain-enhancer of activated B cells (NF-κB) activity driven by IL-17 and concurrently inhibits Caspase 3. Through this mechanism, the IL-17/STEAP4/XIAP signaling axis may contribute to the promotion of carcinogenesis [41].

#### Copper utilization

Upon entering cells, Cu(II) is transported to various intracellular compartments—such as the cytoplasm, mitochondria, Golgi apparatus, and nucleus—by copper chaperones, which facilitate distinct biological functions. The process of copper utilization in humans involves several critical molecular entities. In the cytoplasm, the copper chaperone for copper chaperone for superoxide dismutase (CCS) is essential for delivering copper to target proteins. Specifically, CCS1 activates Cu/Zn superoxide dismutase 1 (SOD1) by transferring Cu(II) to SOD1 in both the cytoplasm and mitochondria, without affecting copper transfer to cytochrome c oxidase (COO) [42]. CCS1 and SOD1, sharing structural similarities, co-localize in the cytoplasm and mitochondrial intermembrane space to combat mitochondrial superoxide [43]. CCS1 binds to an immature form of SOD1, promoting its maturation and enabling SOD1 to guard against radical oxygen species accumulation through the redox cycling of copper at its catalytic center [44]. The aberrant expression of SOD1 has been implicated in various cancers [45].

Mitochondria, critical for ATP production through oxidative phosphorylation, act as significant copper stores and major intracellular users of copper for copper-dependent enzymes [46]. CCS1 within mitochondria serves as a unique import receptor, facilitating SOD1 and CCS1 import and folding, thus broadening the scope of protein import based on oxidation within the mitochondrial intermembrane space [47]. The transport of Cu^2 + to mitochondria is primarily dependent on Cytochrome C oxidase copper chaperone 17 (COX17), which aids in assembling COO by providing Cu(II) for the formation of Cu A/B sites within the enzyme complexes [48]. COX17, along with its homologue COX23, is pivotal for the cytochrome oxidase assembly, transferring Cu(I) from the cytoplasm to mitochondrial membrane proteins synthesis of Cytochrome C oxidase 1 (SCO1) and SCO2 for copper insertion into the mitochondrially encoded SCO2/COX2 [49, 50]. These processes are essential for maintaining mitochondrial copper homeostasis and have implications in cancer development.

In the cytoplasm, the copper chaperone and antioxidant ﻿Antioxidant 1 (ATX1), identified as a ferritin-like protein in yeast, plays a vital role in copper transport pathways and antioxidant functions [51]. The human ATX1 homologue, Antioxidant Protein 1 (ATOX1), acts as a cytoplasmic copper chaperone, transmitting copper to ATPase copper transporting proteins ATP7A and ATP7B within the Golgi network, thus facilitating copper efflux during copper excess [22, 52]. ATOX1 also interacts with cisplatin, influencing cisplatin resistance mechanisms [53]. Copper ions are also directed by CCS into the nucleus to regulate HIF1 activity, with ATOX1 facilitating copper transport into the nucleus in a copper-dependent manner, highlighting its crucial role in cellular copper transport and signaling pathways involved in cancer and other diseases [54].

In addition to the specific roles of copper chaperones in moving and using copper inside cells, metallothioneins also have a key role [3, 55, 56]. These are small proteins rich in cysteine and are crucial in controlling the amount of copper inside cells [57]. Through their capacity to sequester copper ions, these proteins serve a dual function [58–60]. They not only mediate the detoxification of excess copper, thereby averting potential cytotoxicity, but also facilitate the strategic accumulation of a bioavailable copper pool. This dual functionality underscores the pivotal role of metallothioneins in the maintenance of copper homeostasis, safeguarding cellular integrity against copper-induced oxidative stress while ensuring the requisite availability of this trace element for critical enzymatic processes [61, 62]. The integration of metallothioneins into the cellular framework for managing copper further illustrates the complexity of copper homeostasis and its significance in cellular health and disease pathogenesis.

#### Copper export

Copper efflux transporters ATP7A and ATP7B play pivotal roles in mediating the release of Cu(II) into the secretory pathway and facilitating its export to the extracellular environment, thus maintaining cellular copper homeostasis [21]. Mutations in these transporters are linked to Menkes and Wilson’s diseases, highlighting their crucial role in human health. The redox modulation of ATP7A and ATP7B is mediated by glutathione (GSH) and glutaredoxin1 (GRX1), which regulate copper binding through the Cu-ATPase cysteine motif, ensuring the proper functioning of these transporters [63, 64]. Additionally, Clusterin and copper metabolism domain containing 1 (COMMD1) provide alternative mechanisms for the quality control of ATP7A and ATP7B, promoting their degradation through independent pathways and facilitating their recycling between the endosomal network and the cell membrane via specific molecular complexes, including COMMD/CCDC22/CCDC93 [65, 66].

**Fig. 3:**
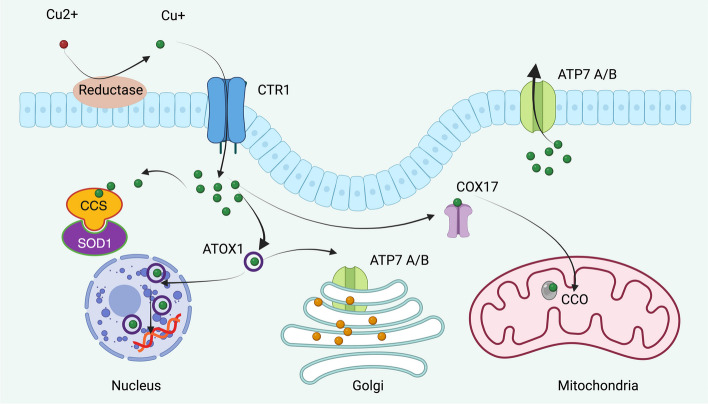
Intracellular copper distribution pathways. The intracellular copper metabolism signaling pathway involves a sequence of events starting with the reduction of extracellular Cu(II) to Cu(I), which is then transported into the cell via the CTR1. Once inside, Cu(I) associates with the copper chaperone for CCS and SOD1, facilitating its distribution to various subcellular compartments. Additionally, Cu(I) interacts with ATOX1, predominantly directing it to ATP7A and ATP7B, for transport to the Golgi apparatus. A minor portion of Cu(I) binds to transcription factors in the nucleus, influencing gene expression. Cu(I) also binds to COX17 and is transported to CCO in mitochondria, contributing to the oxidative phosphorylation process. Surplus Cu(I) is expelled from the cell through ATP7A/B on the plasma membrane

The N-terminal region of ATP7B contains six metal-binding domains, each featuring a copper-binding motif, MXCXXC, which are crucial for the activity of ATP7B and its copper-induced intracellular redistribution [67]. The dynamics of this regionare enhanced upon copper binding, indicating its significance in the function of the transporter [68]. ATOX1 directly interacts with the N termini of ATP7A and ATP7B in a copper-dependent manner, essential for transport of copper to the secretory pathway. Distinctly, the dynactin subunit p62 shows a preference for interacting with the N terminus of ATP7B in the presence of copper, differing from its interaction with ATP7A [69] (Fig. 3).

The COMMD family, encompassing ten members (COMMD1–COMMD10), is vital in copper metabolism, with COMMD1 involved in vesicular copper sequestration [70]. Its downregulation is implicated in tumor progression and the enhancement of inflammatory and stemness characteristics of cancer cells. Copper has been identified as a factor contributing to the radioresistance observed in hepatocellular carcinoma (HCC) [71, 72]. Ionizing radiation decreases COMMD10 expression, leading to increased intracellular copper levels and radioresistance in HCC. This downregulation also affects the ubiquitination degradation pathway of hypoxia-inducible factor 1-alpha (HIF1α), enhancing its nuclear translocation and the transcription of ceruloplasmin and SLC7A11, thereby inhibiting cell death. Elevated ceruloplasmin levels further augment HIF1α expression by reducing iron availability, illustrating how COMMD10 attenuates the HIF1α/ceruloplasmin (HIF1α/CP) feedback loop to enhance cell death and radiosensitivity by disrupting the copper-iron balance [73, 74].

### Role of copper dysregulation in cancer development

Cuproptosis has recently delineated as a cellular demise pathway initiated by copper [75]. Cuproptosis is intricately linked to mitochondrial functions and plays a pivotal role in the dynamics of tumor progression, including cell proliferation, metastasis, and resistance to chemotherapy [76, 77]. Elevated or altered copper levels in the serum and tumor tissues have been identified in various cancers, including breast [78], pancreatic [79], thyroid [80], leukemia [81], colorectal [82], lung [83], prostate [84], and oral cancers [85], suggesting a significant impact on tumorigenesis, invasiveness, and chemoresistance [86]. Notably, the influence of copper extends to modulating HIF1α levels, thereby promoting angiogenesis and vascular endothelial growth factor production [54]. Mediator of cell motility 1 (MEMO1) has been implicated in breast cancer metastasis through its interaction with copper ions and subsequent ROS production, although recent evidence suggests it binds more readily to Cu(I), potentially mitigating redox activity [87, 88].

Copper ions are also believed to be intricately involved in various signaling pathways within tumor cells by interacting and activating key molecules. Copper plays a crucial role in receptor tyrosine kinase (RTK)-related pathways, where it can bind and phosphorylate RTKs without the need for ligand binding, leading to RTK activation. This activation triggers the phosphorylation of downstream extracellular regulated protein kinases (ERK) and agammaglobulinaemia tyrosine kinase (ATK), ultimately promoting cell migration and proliferation [89]. Additionally, copper ions influence the phosphoinositide-3-kinase (PI3K)-AKT signaling pathway. Copper can directly activate PI3K, resulting in subsequent AKT activation. Furthermore, copper binds to specific sites on pyruvate dehydrogenase kinase 1 (PDK1)—histidine117 and histidine203—leading to AKT activation[90]. This activation of AKT by copper catalyzes the phosphorylation and subcellular redistribution of forkhead box O1a (FoxO1a) and forkhead box O4 (FoxO4), thereby fostering cancer cell proliferation and tumor growth [91, 92].

The activation of the mitogen-activated protein kinase (MAPK) signaling pathway is also copper-dependent [93]. Copper directly binds to mitogen-activated protein kinase kinase 1 (MEK1), promoting the phosphorylation of ERK1/2 and activating the downstream c-Jun N-terminal kinase (JNK) to regulate tumor growth [94]. This highlights the multifaceted role of copper in modulating key signaling pathways within tumor cells, impacting critical processes like cell proliferation, migration, and overall tumor development. Copper ions play a pivotal role in multiple cancer signaling pathways, impacting tumor cell behavior and proliferation. The autophagy pathway, which recycles metabolic waste to meet energy needs or evade apoptosis in tumor cells, is directly influenced by copper. Copper binds to Unc-51 Like autophagy activating kinase (ULK) and serves as a regulator, promoting the phosphorylation and activation of autophagy-related 13 (ATG13) [95, 96]. This leads to the formation of the autophagic complex and consequent tumor growth. Similar to the MAPK pathway, the autophagy pathway, influenced by copper, supports cancer cell survival.

In lung adenocarcinoma cells driven by the BRAF, a reduction in copper ion concentration, due to the loss of CTR1, correlates with decreased activity of MEK1/2 and ULK1/2, essential kinases in these pathways [97]. Copper ions also affect the biological behavior of cancers indirectly through various pathways. For instance, in the Notch pathway, typically a tumor suppressor, copper promotes the shedding of the notch ligand Jagged1, enhancing tumor cell migration [98–101].

The relationship of copper with tumor angiogenesis is significant, particularly through its interaction with HIF-1α-related pathways. Copper mediates the binding of HIF-1α to target gene promoters, even under normoxic conditions, stabilizing HIF-1α and promoting expression of genes like VEGF, which drives tumor angiogenesis [3, 54, 102, 103]. Furthermore, copper interacts with the Nuclear factor kappa-light-chain-enhancer of activated B cells (NFκB) pathway, influencing inflammation and tumorigenesis. Elevated intracellular copper levels, induced by inflammatory cytokines, activate the XIAP, which in turn activates NFκB [104]. Additionally, copper's role in lipolysis pathways has been highlighted, particularly in its interaction with the Wnt signaling pathway, which regulates cell renewal and exhibits pro-tumor effects in tumor cells. The impact of copper on the Wnt pathway-realted key molecules, β-catenin and C-myc, is debated, with studies showing copper can both decrease and increase C-myc stability through phosphorylation [105].

Copper also regulates tumor metabolism, impacting lipid and sugar metabolic pathways. It interacts with phosphodiesterase 3B (PDE3B) to regulate lipolysis and is thought to suppress tumor growth by inhibiting the expression of ribosomal protein S6 kinase beta-1 (S6K1) and downstream glycolysis-related molecules, including Glucose transporter 1 (GLUT1), pyruvate kinase M2 (PKM2), and lactate dehydrogenase A (LDHA) [106, 107]. In summary, copper's direct and indirect roles in various cancer signaling pathways underline its critical importance in the development and progression of cancers.

### Molecular mechanisms of cuproptosis

Over recent years, the relationship between copper and cell death has garnered considerable focus, particularly the mechanisms through which copper triggers cell demise. Since the 1980s, the capacity of copper to induce cell death has been recognized, yet the detailed mechanisms remained undefined [108]. Understanding the molecular orchestration of cuproptosis unveils significant pathways through which copper instigates cellular demise. This section delineates the sequential molecular events precipitated by copper, emphasizing the pivotal roles of Ferredoxin 1 (FDX1), ﻿dihydrolipoamide S- acetyltransferase (DLAT), and lipoylation-related proteins in orchestrating cuproptosis. By dissecting these intricate processes, we aim to illuminate the underlying mechanisms that render cuproptosis a promising target for innovative cancer therapeutics.

**Fig. 4:**
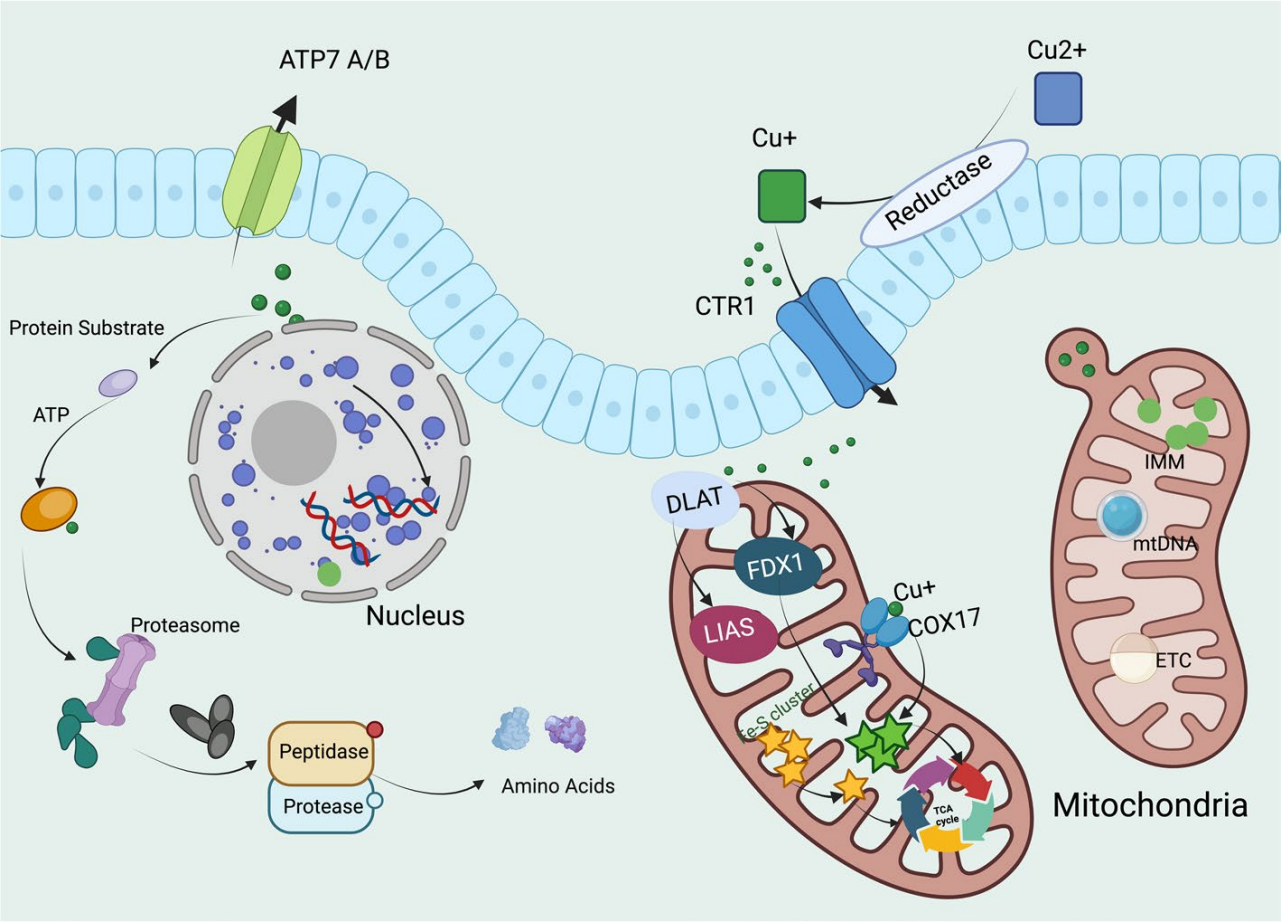
Molecular mechanisms of cuproptosis. The molecular mechanisms underlying cuproptosis involve several key processes facilitated by copper ions. i. Cu(II) outside the cell are reverted to Cu(I) by a reductase enzyme. This oxidization-reduction form of copper is then transported into the cell by CTR1. ii. Copper is handled by various chaperone proteins to prevent toxic accumulation inside cell. ATP7A/B can pump excess copper out of the cell or deliver it to the Golgi apparatus for incorporation into copper-dependent enzymes. iii. Copper plays an essential role in several biochemical pathways inside the mitochondria. Here, it is involved in the ETC, which is a series of protein complexes embedded in the inner mitochondrial membrane. iv. The copper chaperone COX17 delivers copper to the mitochondrial enzyme complexes, such as COO for their proper function. The mitochondrion matrix contains several enzymes, including LIAS and DLAT, which are part of the TCA cycle. v. FDX1 is involved in the transfer of electrons in metabolic processes and is believed to interact with copper ions directly or indirectly affecting the TCA cycle. vi.the presence of mitochondrial DNA suggests that changes in copper homeostasis might impact mitochondrial genetics and, consequently, the function of organelle. Abbreviation: ETC, electron transport chain; IMM, inner mitochondrial membrane; mtDNA, mitochondrial DNA; LIAS, Lipoic Acid Synthase;DLAT; Dihydrolipoamide S-Acetyltransferase

Cuproptosis is triggered by the direct interaction between copper ions and lipoacylated components within the TCA cycle, leading to protein aggregation and cellular stress. This interaction primarily involves proteins like FDX1 and DLAT that play critical roles in the cellular copper homeostasis and metabolic pathways. More and more evidence have supported the critical involvement of FDX1 and DLAT in cuproptosis [109]. For instance, targeted deletion of FDX1 in murine models significantly mitigates cuproptosis, underscoring its indispensability[110]. Similarly, pharmacological inhibition of DLAT lipoylation capacity has been shown to arrest the cuproptosis process, offering therapeutic insights [1]. FDX1 acts as a linchpin in the copper-mediated disruption of iron-sulfur clusters, destabilizing mitochondrial function. The susceptibility of DLAT to copper perturbation highlights the vulnerability of metabolic processes to copper overload. The alteration in lipoylation underscores the specificity of the cytotoxicity of copper, delineating a pathway distinct from conventional apoptosis or necrosis. The role of copper ionophores, which are lipid-soluble molecules binding copper ions reversibly, became pivotal in uncovering cuproptosis, potentially influencing antitumor therapy [111]. These ionophores facilitate copper transport across cellular and mitochondrial membranes. DSF, traditionally used for alcohol dependency, acts as a copper ionophore implicated in cell death, similar to elesclomol (ES), another ionophore believed to possess cytotoxic capabilities [112, 113]. Investigations into ES and DSF have led researchers to propose that copper itself, rather than ionophores, is responsible for this cell death, despite unclear mechanisms [114]. ROS, primarily produced by mitochondria, align with the linkage between copper-induced cell death and mitochondrial metabolism [115]. Studies agree that ES-induced cell death results from elevated ROS levels, attributed to various mitochondrial factors. In melanoma and leukemia cell lines, copper was shown to enhance ROS production, affecting cell proliferation and mitochondrial functions [114]. The discovery of FDX1 as crucial for ES sensitivity, directly interacting with ES-Cu and inhibiting iron-sulfur cluster formation, marked a significant advance [116]. Further research in glioblastoma cells demonstrated that ES-Cu directly impacts mitochondrial membrane potential, an effect modifiable by tetrathiomolybdate (TTM) [117]. Similarly, DSF transports copper into cells, impacting the mitochondrial respiratory chain and ROS levels [118]. The interaction between DSF-Cu and Npl4 is also linked to copper-induced cell death, suggesting the role of copper in inhibiting protein degradation pathways [119–121]. While past research predominantly associated copper-induced cell death with mitochondrial ROS production, recent findings challenge this notion, indicating the direct involvement of copper beyond known cell death pathways like apoptosis, autophagy, or ferroptosis. The identification of cuproptosis by *Tsvetkov *et al*.* as a distinct cell death mechanism driven by copper, independent of traditional apoptotic or ferroptotic pathways, represents a groundbreaking shift in our understanding of the cytotoxic effects of copper [1](Fig. 4)*.*

The reduction in cell death triggered by ES upon diminishing its copper-binding capability, and its complete cessation with the removal of this functionality, underscores the independence of copper ions in inducing cellular demise without the involvement of ES [116]. Beyond ES and DSF, additional ionophores mirror these cellular outcomes [1]. GSH, a natural intracellular copper chelator, elevates copper levels within cells, leading to death [1]. Pertinently, in models related to Wilson's Disease, a disruption in copper equilibrium via ATP7B downregulation has also been implicated in inducing cell death [22].

Cell death induced by copper ionophores, classified independently from traditional cell death mechanisms, introduces cuproptosis [1]. This process involves interactions with the components of TCA cycle within mitochondria, highlighting a conserved lipoylation pathway. Investigations to pinpoint the precise mitochondrial respiration components affected by copper utilized inhibitors like Oligomycin, FCCP (Trifluoromethoxy carbonylcyanide phenylhydrazone), and antimycin A/rotenone, revealing a significant impact on the spare capacity of respiration, thus indicating the direct interaction of copper with the TCA cycle rather than with the electron transport chain (ETC) or adenosine triphosphate (ATP) synthesis components [1]. Genomic, metabolic, and individual gene knockouts have identified crucial genes for cuproptosis, classifying them into categories: FDX1, genes associated with the lipoic acid (LA) pathway (LIAS and LIPT1), and those part of the pyruvate dehydrogenase complex (PDC) crucial for mitochondrial respiration (DLAT; DLD: dihydrolipoamide dehydrogenase; PDHA1: pyruvate dehydrogenase E1 alpha 1 subunit; PDHB: pyruvate dehydrogenase E1 beta subunit), all linked to the LA pathway [122]. FDX1, potentially upstream in the LA pathway, aids in attaching the lipoyl group to DLAT, vital for the mitochondria the functionality of PDC [122].

The status of copper metabolism-related genes and proteins, such as the expression levels of FDX1 and DLAT, could serve as predictive markers for the efficacy of cuproptosis-inducing therapy. Alterations in the expression or function of these proteins might indicate the cellular copper status and susceptibility to cuproptosis, providing a basis for personalized therapeutic approaches. However, despite advancements in delineating cuproptosis mechanisms through specific cell and mouse models by *Tsvetkov* et al. [1], significant questions remain, particularly regarding the characteristic manifestations of cuproptosis, the downstream effects of DLAT oligomerization, and the comprehensive role of FDX1. These gaps underscore the need for further exploration to fully comprehend cuproptosis and its potential implications in disease pathogenesis and therapy, advocating for a collaborative effort to transcend current research paradigms and embrace innovative therapeutic strategies.

### Cuproptosis and other known cell death pathways

**Fig. 5:**
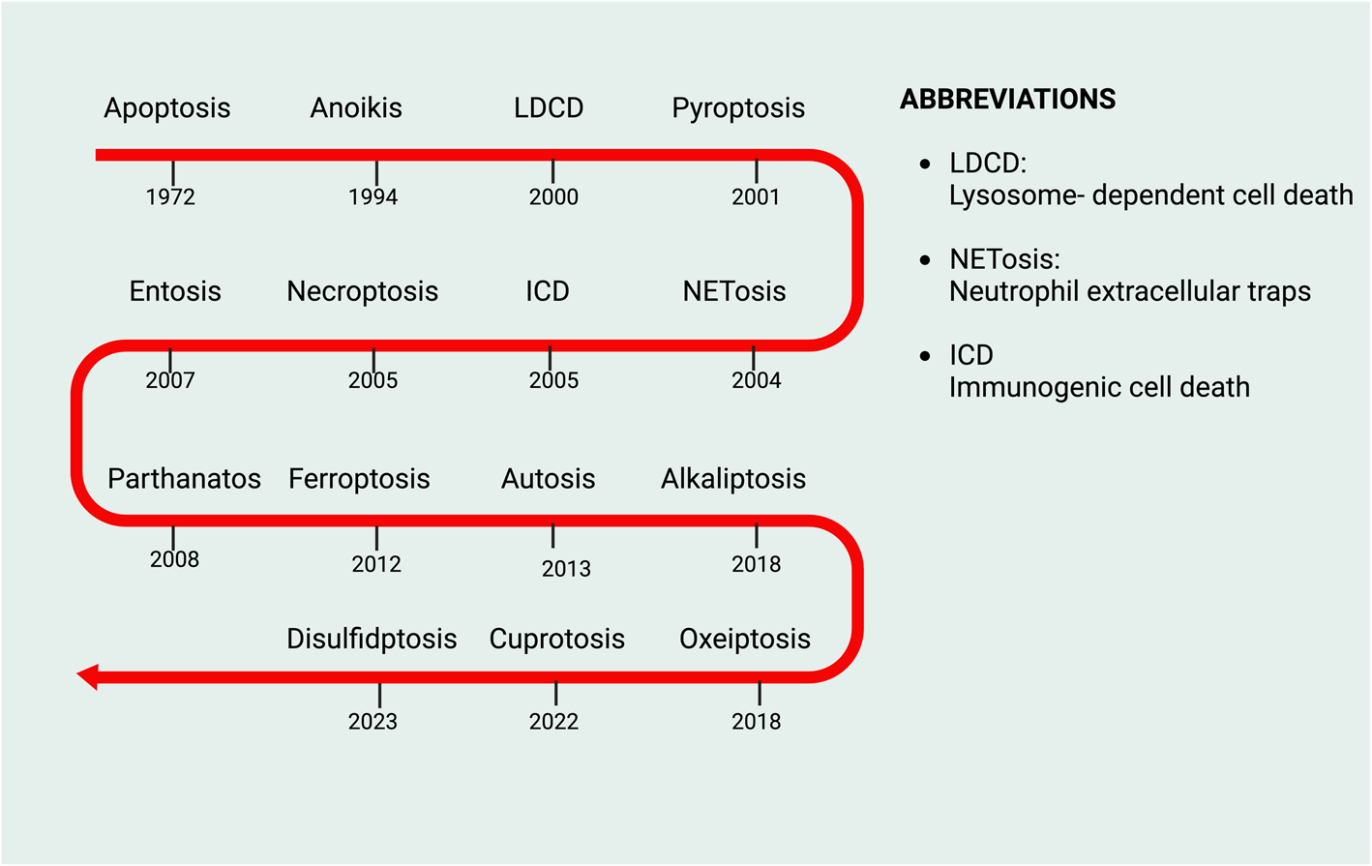
Timeline of key discoveries related to cell death. The exploration of cell death mechanisms has unveiled numerous modes beyond traditional necrosis, each with distinct triggers and cellular responses. Apoptosis, identified in the 1970s, emerged as the first form of programmed cell death, pivotal for development and disease. The 2000s introduced autophagy and necroptosis, highlighting the complexity of cell death and its role in health and pathology. Discoveries like cuproptosis in recent years have further expanded our understanding, revealing the intricate balance cells maintain between survival and death. These advancements underscore the diverse cellular strategies for self-destruction, critical for therapeutic targeting in diseases. Abbreviation: LDCD, lysosome-dependent cell death; NETosis, neutrophil extracellular traps; ICD, immunogenic cell death

While the intricate dance between necroptosis, pyroptosis, ferroptosis, and cuproptosis highlights the complexity of cellular death mechanisms, we suggests that cuproptosis, in particular, represents a unique vulnerability of cancer cells. This vulnerability, rooted in the distinct molecular pathways of cuproptosis, could offer a new therapeutic target that is yet to be fully exploited in cancer treatment strategies (Fig. 5). Research has shown that necroptosis can trigger the Nucleotide- binding oligomerization domain, leucine- rich repeat and pyrin domain- containing 3 (NLRP3) inflammasome activation by facilitating potassium release through mixed lineage kinase domain-like protein (MLKL)-formed pores in macrophages [123]. The Z-DNA-binding protein 1 (ZBP1, also known as DNA-dependent activator of IFN-regulatory factors protein), recognizing viral or endogenous nucleic acid ligands, initiates an innate immune response, leading to the recruitment of Receptor Interacting Protein Kinase 3 (RIPK3) and caspase-8, which, in turn, activates the NLRP3 inflammasome, thus prompting both necroptosis and pyroptosis [124–127]. Furthermore, bioinformatics analysis by *Miao *et al*.* suggests that ZBP1, related to both cuproptosis and necroptosis, could serve as a prognostic indicator for low-grade glioma patients [128]. *Wei Gao* research found that treating colorectal cancer (CRC) cells with ES raises mitochondrial Cu(II) levels while reducing ATP7A expression, culminating in ROS build-up. This action promotes SLC7A11 degradation, enhancing oxidative stress and leading to ferroptosis in CRC cells [129]. Additionally, a novel glucose oxidase (GOx)-engineered nonporous Cu(I) 1,2,4-triazolate ([Cu(tz)]) coordination polymer nanodrug, GOx@[Cu(tz)], effectively merges cancer starvation therapy with cuproptosis induction [7]. This catalytic activity of nanodrug is activated in the presence of high GSH levels within cancer cells, depleting glucose [130]. The ensuing redox reaction between released Cu(II) and intracellular GSH causes GSH depletion and reduces Cu(II) to Cu(I), the latter acting as a Fenton agent to produce hydroxyl radicals via the Fenton reaction, thereby predisposing cancer cells to cuproptosis and potentially ferroptosis due to glucose and GSH depletion [7].

Ferroptosis has emerged prominently over the past decade as a notable form of regulated cell death, characterized by oxidative disturbances within cells, mainly due to excessive iron and lipid peroxidation [131]. This particular form of cell death, distinct from apoptosis, garnered attention in 2003 with the discovery that erastin, a permeable compound identified through high-throughput screening, selectively targeted cells harboring oncogenic RAS mutations for death. The formal adoption of the term "Ferroptosis" in 2012 helped categorize this iron-dependent, non-apoptotic death pathway [131]. Notable morphological changes during ferroptosis include alterations in mitochondria, such as decreased cristae, denser membranes, and rupture, alongside lipid peroxidation, increased ROS, and the modulation of ferroptosis-specific genes [132–134].

**Fig. 6:**
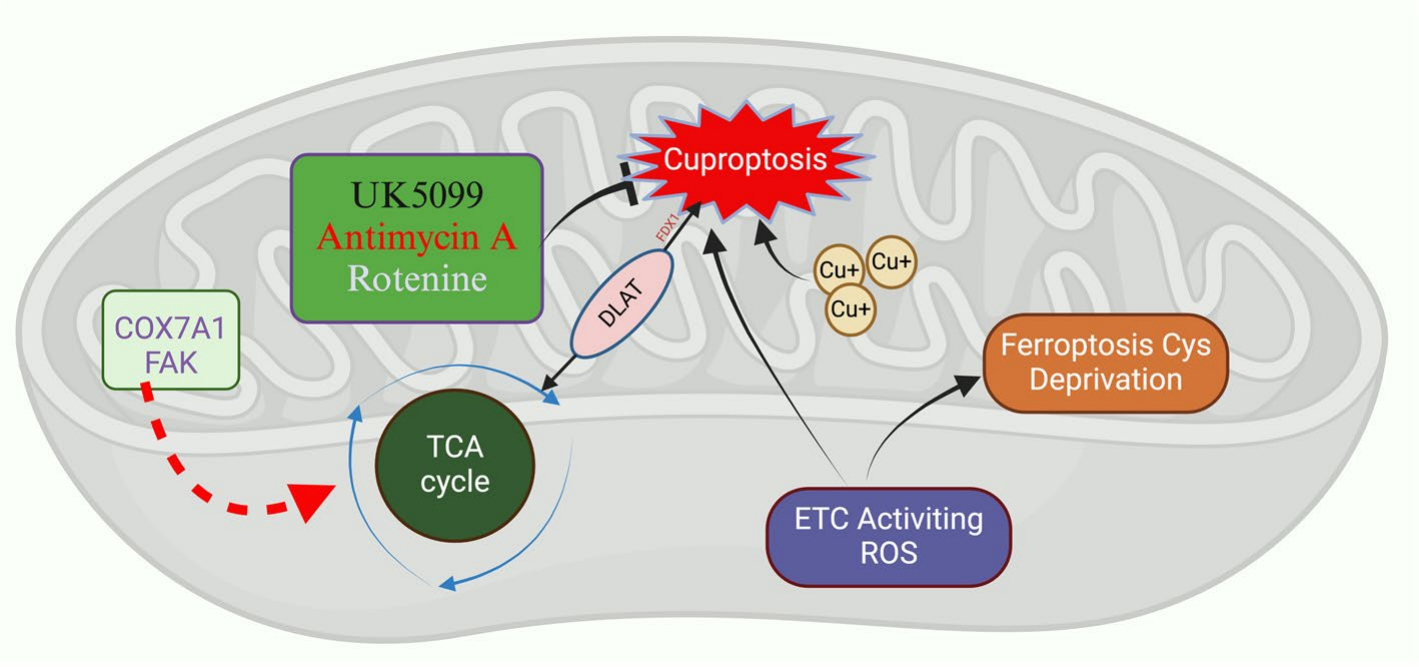
The crosstalk between ferroptosis and cuproptosis inside mitochondria. TCA cycle within mitochondria acts as a co-regulator for both cystine-depleted ferroptosis and cuproptosis. The enzymes COX7A1 and FAK are shown to amplify the mitochondrial TCA cycle, thereby enhancing production and promoting the onset of both cystine-depleted ferroptosis and cuproptosis. Cuproptosis is depicted as dependent on mitochondrial respiratory activities and is shown to be inhibited by specific mitochondrial respiration inhibitors such as rotenone, antimycin, and UK5099. Abbreviation: COX7A1, cytochrome c oxidase subunit 7A1; FAK, focal adhesion kinase; DLAT, dihydrolipoamide s-acetyltransferase; etc., electron transport chain. Created with BioRender

Biological investigations have established a significant association between ferroptosis and cuproptosis in cancer progression (Table 1 & Fig. 6)*.* In hepatocellular carcinoma, an amalgamation of genes related to both cuproptosis and ferroptosis has been shown to have prognostic value, predicting responses to immunotherapy and sensitivities to various drugs [135]. Similarly, in lung adenocarcinoma, the interaction between regulators of cuproptosis and ferroptosis delineates the tumor microenvironment (TME), influencing chemotherapy outcomes and overall prognoses [136]. In breast cancer, a correlation between genes associated with cuproptosis and ferroptosis has been observed with immune cell infiltration and patient survival rates [137].

**Table 1:** Comparison between Ferroptosis and Cuproptosis

Features	Ferroptosis	Cuproptosis
Conditions	Iron accumulation and lipid peroxidation	Copper accumulation and mitochondrial respiration
Inhibition	Antioxidants like SLC7A11, GPX4, and Ferrostatins	Inhibitors of mitochondrial respiration (e.g., Rotenone, Antimycin)
Mitochondrial Involvement	Yes	Yes
Metabolic Pathway Involved	Lipid peroxidation pathways	TCA cycle
Key Enzymes/Proteins	GPX4, ACSL4, LPCAT3	COX7A1, FDX1, FAK
Associated Diseases	Neurodegenerative diseases, cancer, kidney failure	Not specified, research ongoing
Cellular Outcome	Cell death due to peroxidation damage	Cell death due to mitochondrial dysfunction

*Abbreviation:* *SLC7A11* ﻿Solute carrier family 7 member 11, *GPX4* ﻿Gluta- thione peroxidase 4, *ACSL4* ﻿Long-chain acyl-CoA synthetase-4, *LPCAT3* ﻿Lysophosphatidylcholine acyltransferase 3, *TCA* Tricar- boxylic acid cycle, *COX7A1* ﻿Cytochrome c oxidase subunit 7A1, *FDX1* ﻿Ferredoxin, *FAK* ﻿Focal adhesion kinase

Further investigations prior to the formal introduction of "cuproptosis" as a term suggested a link between excessive copper accumulation and ferroptosis [129]. Copper overload, induced by the cuproptosis inducer ES, triggers ferroptosis in CRC. This process begins by increasing oxidative stress. As a result, copper accumulates in the mitochondria, leading to the generation of ROS. These ROS then lead to the degradation of SLC7A11.This outlines the co-regulatory role of ES in both cuproptosis and ferroptosis pathways [129]. Moreover, the copper ionophore DSF, alongside a copper complex, has exhibited anticancer properties by disrupting mitochondrial homeostasis and inducing ferroptosis, thereby inhibiting migration and invasion in liver cancer cells [138].

Notably, copper ionophores have been identified to mediate ferroptosis in various cancer models, highlighting the potential anticancer effects of copper overload through ferroptosis. Prior to recognizing cuproptosis, it was widely accepted that intracellular copper accumulation could escalate ROS levels via the Fenton reaction or mitochondrial damage, culminating in lipid peroxidation of cell membranes and ferroptosis [3]. With the identification of cuproptosis, a unique copper-dependent cell death mechanism, the anticancer effects of ES/DSF in colorectal and liver cancer cells are speculated to involve mechanisms of both ferroptosis and cuproptosis [129]. The dysregulation of copper homeostasis appears to influence not just cuproptosis but also ferroptosis, warranting further experimental validation [129].

**Fig. 7:**
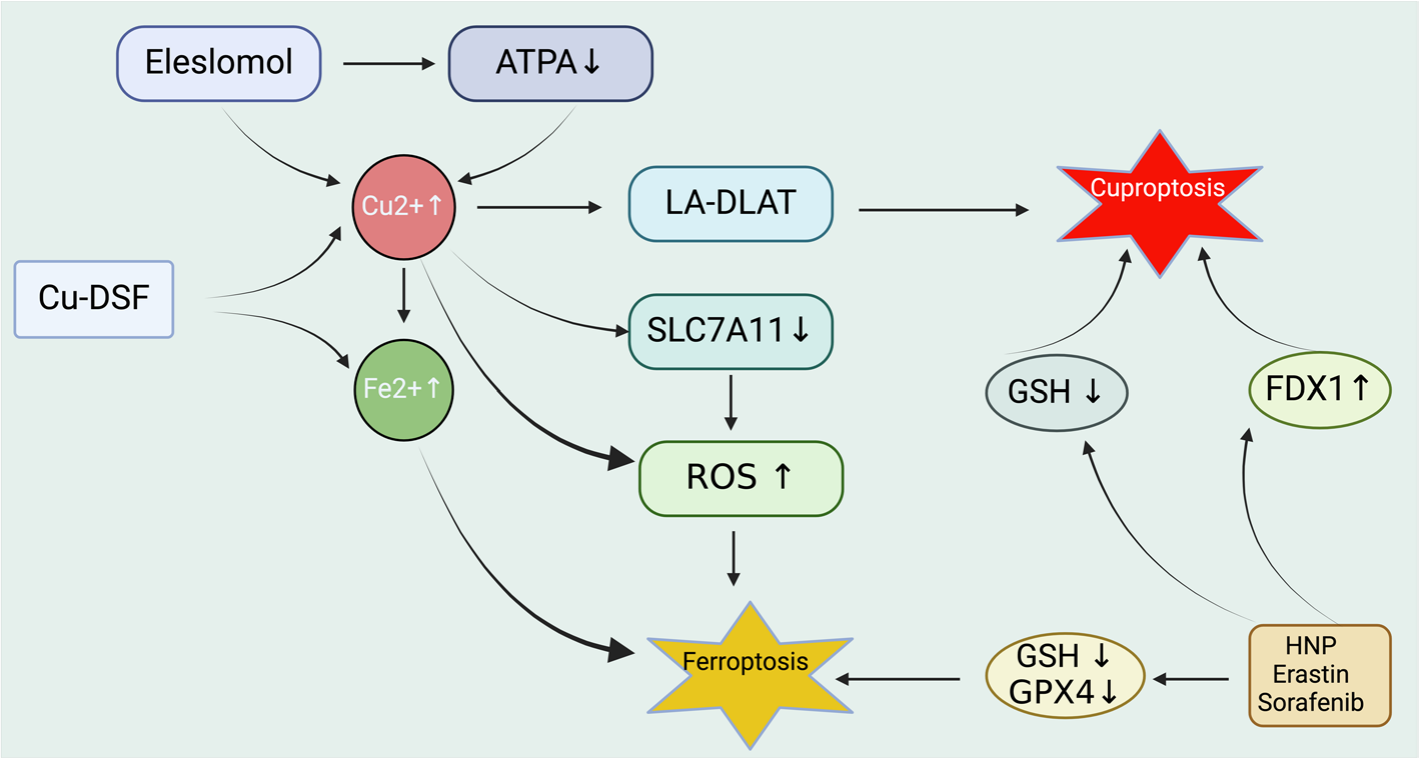
The crosstalk between ferroptosis and cuproptosis mechanisms in cancer. ES increases intracellular Cu(II) concentration and inhibits ATP7A, thus contributing to cuproptosis. Concurrently, Cu-DSF complex elevates Cu(II) and Fe(II) levels, which would lead to an increase in ROS. Increased Cu (II) impairs LA-DLAT, which is linked to cuproptosis, while decreased expression of SLC7A11 reduces GSH levels. This increase in ROS, stemming from both the elevation of Fe (II) and the decrease in SLC7A11, would trigger ferroptosis, which is a form of cell death characterized by lipid peroxidation due to iron accumulation. A decrease in GSH and GPX4 also further drives the cell towards ferroptosis, while levated FDX1 levels facilitate cuproptosis. Abbreviation:ATPA, adenosine triphosphatase; HNP, hollow nano platform. Created with BioRender

Recent studies on liver cancer have revealed that inducers of ferroptosis, like sorafenib and erastin, can impact the process of cuproptosis [139]. These inducers work by preventing the degradation of the FDX1 protein. Additionally, they reduce the synthesis of intracellular GSH. This reduction in GSH synthesis, along with the prevention of FDX1 degradation, promotes the aggregation of lipoylated proteins, which ultimately leads to cuproptosis [129]. The combined application of sorafenib and ES has demonstrated enhanced efficacy in inhibiting both cancer cell growth and tumor proliferation, underscoring the intricate interplay between ferroptosis and cuproptosis in cancer treatment strategies (Fig. 7).

Current insights reveal that ferroptosis and cuproptosis significantly contribute to the infiltration of immune cells within the TME [140, 141]. Despite their potential dual roles of exhibiting both synergistic and antagonistic effects during immunotherapeutic interventions, understanding the interplay between the TME and these regulated mechanisms of cell death could provide strategies to overcome the challenge of insufficient immunotherapy responses observed in a subset of patients. The enhancement of anti-cancer outcomes through the stimulation of cuproptosis and ferroptosis alongside chemotherapy and immunotherapy has been documented [142, 143]. While radiotherapy is known to trigger ferroptosis, enhancing tumor cell sensitivity to such treatment, the potential induction of cuproptosis by radiotherapy is yet to be elucidated [144, 145]. The relationship between cuproptosis and ferroptosis within the context of cancer therapy is contingent upon the specific treatment modalities and the intrinsic nature of the targeted malignancies. The intricate regulatory interconnection between these two cell death pathways suggests possible variable impacts under differing conditions. Joint targeting of both cuproptosis and ferroptosis in anticancer treatment strategies has demonstrated superior efficacy over single-target approaches in both in vitro and in vivo models, albeit primarily within a singular cancer type context [146]. Given the diversity of tumor types and the intricate nature of the TME, further exploration into the unidentified molecular underpinnings is critical. Additionally, developing strategies to minimize harm to healthy tissues from cuproptosis and ferroptosis necessitates a comprehensive approach involving extensive preclinical and clinical investigations, as well as the innovation of new therapeutic modalities and technologies. The advent of nanomedicine may offer a promising avenue in this realm [143, 147–152]. Considering the present scenario, there is a strong advocacy for detailed research exploring the dynamics between ferroptosis, cuproptosis, and cancer to inform and refine anticancer treatment approaches that effectively co-target these pathways of regulated cell death.

### Role of copper dysregulation in cancer development

Cuproptosis has recently delineated as a cellular demise pathway initiated by copper [75]. Cuproptosis is intricately linked to mitochondrial functions and plays a pivotal role in the dynamics of tumor progression, including cell proliferation, metastasis, and resistance to chemotherapy [76, 77]. Elevated or altered copper levels in the serum and tumor tissues have been identified in various cancers, including breast [78], pancreatic [79], thyroid [80], leukemia [81], colorectal [82], lung [83], prostate [84], and oral cancers [85], suggesting a significant impact on tumorigenesis, invasiveness, and chemoresistance [86]. Notably, the influence of copper extends to modulating HIF1α levels, thereby promoting angiogenesis and vascular endothelial growth factor production [54]. Mediator of cell motility 1 (MEMO1) has been implicated in breast cancer metastasis through its interaction with copper ions and subsequent ROS production, although recent evidence suggests it binds more readily to Cu(I), potentially mitigating redox activity [87, 88].

Copper ions are also believed to be intricately involved in various signaling pathways within tumor cells by interacting and activating key molecules. Copper plays a crucial role in receptor tyrosine kinase (RTK)-related pathways, where it can bind and phosphorylate RTKs without the need for ligand binding, leading to RTK activation. This activation triggers the phosphorylation of downstream extracellular regulated protein kinases (ERK) and agammaglobulinaemia tyrosine kinase (ATK), ultimately promoting cell migration and proliferation [89]. Additionally, copper ions influence the phosphoinositide-3-kinase (PI3K)-AKT signaling pathway. Copper can directly activate PI3K, resulting in subsequent AKT activation. Furthermore, copper binds to specific sites on pyruvate dehydrogenase kinase 1 (PDK1)—histidine117 and histidine203—leading to AKT activation[90]. This activation of AKT by copper catalyzes the phosphorylation and subcellular redistribution of forkhead box O1a (FoxO1a) and forkhead box O4 (FoxO4), thereby fostering cancer cell proliferation and tumor growth [91, 92].

The activation of the mitogen-activated protein kinase (MAPK) signaling pathway is also copper-dependent [93]. Copper directly binds to mitogen-activated protein kinase kinase 1 (MEK1), promoting the phosphorylation of ERK1/2 and activating the downstream c-Jun N-terminal kinase (JNK) to regulate tumor growth [94]. This highlights the multifaceted role of copper in modulating key signaling pathways within tumor cells, impacting critical processes like cell proliferation, migration, and overall tumor development. Copper ions play a pivotal role in multiple cancer signaling pathways, impacting tumor cell behavior and proliferation. The autophagy pathway, which recycles metabolic waste to meet energy needs or evade apoptosis in tumor cells, is directly influenced by copper. Copper binds to Unc-51 Like autophagy activating kinase (ULK) and serves as a regulator, promoting the phosphorylation and activation of autophagy related 13 (ATG13) [95, 96]. This leads to the formation of the autophagic complex and consequent tumor growth. Similar to the MAPK pathway, the autophagy pathway, influenced by copper, supports cancer cell survival.

In lung adenocarcinoma cells driven by the BRAF, a reduction in copper ion concentration, due to the loss of CTR1, correlates with decreased activity of MEK1/2 and ULK1/2, essential kinases in these pathways [97]. Copper ions also affect the biological behavior of cancers indirectly through various pathways. For instance, in the Notch pathway, typically a tumor suppressor, copper promotes the shedding of the notch ligand Jagged1, enhancing tumor cell migration [98–101].

The relationship of copper with tumor angiogenesis is significant, particularly through its interaction with HIF-1α-related pathways. Copper mediates the binding of HIF-1α to target gene promoters, even under normoxic conditions, stabilizing HIF-1α and promoting expression of genes like VEGF, which drives tumor angiogenesis [3, 54, 102, 103]. Furthermore, copper interacts with the Nuclear factor kappa-light-chain-enhancer of activated B cells (NFκB) pathway, influencing inflammation and tumorigenesis. Elevated intracellular copper levels, induced by inflammatory cytokines, activate the XIAP, which in turn activates NFκB [104]. Additionally, copper's role in lipolysis pathways has been highlighted, particularly in its interaction with the Wnt signaling pathway, which regulates cell renewal and exhibits pro-tumor effects in tumor cells. The impact of copper on the Wnt pathway-realted key molecules, β-catenin and C-myc, is debated, with studies showing copper can both decrease and increase C-myc stability through phosphorylation [105].

Copper also regulates tumor metabolism, impacting lipid and sugar metabolic pathways. It interacts with phosphodiesterase 3B (PDE3B) to regulate lipolysis and is thought to suppress tumor growth by inhibiting the expression of ribosomal protein S6 kinase beta-1 (S6K1) and downstream glycolysis-related molecules, including Glucose transporter 1 (GLUT1), pyruvate kinase M2 (PKM2), and lactate dehydrogenase A (LDHA) [106, 107]. In summary, copper's direct and indirect roles in various cancer signaling pathways underline its critical importance in the development and progression of cancers.

### Cuproptosis ﻿in cancer proliferation and metastasis

Cuproptosis has been identified as potentially inhibiting the proliferation of cancer cells and preventing the spread of metastases. For example, exposure to arecoline from betel nut consumption in oral squamous cell carcinoma cases may impede cuproptosis, thus enhancing the survival of cancer-associated fibroblasts [153]. Cancer-associated fibroblasts play a pivotal role in the advancement of cancer by fostering epithelial-mesenchymal transition, metastasis, and resistance to chemotherapy [154, 155]. Furthermore, tumors undergoing cuproptosis exhibit reduced angiogenesis and display sensitivity towards the treatments with sunitinib and sorafenib [156]. Intriguingly, cancer cells have developed strategies to circumvent copper-induced apoptosis, ensuring their continued survival. A significant revelation by Zhang et al*.* was the substantial downregulation of FDX1, a crucial regulator of cuproptosis, in patients with HCC, which contributes to the resistance of HCC cells to cuproptosis [157]. Additionally, lower levels of FDX1 expression have been linked to more severe stages of tumor-node-metastasis [158] and associated with diminished survival rates in various types of cancer [157, 159, 160]. Despite these observations, the occurrence of cuproptotic events across a broad spectrum of cancers remains inadequately documented. Consequently, further research through both in vitro and in vivo studies is essential to substantiate the impact of cuproptosis on the growth and spread of cancer cells.

### Cuproplasia and cuproptosis

Cuproplasia represents a newly conceptualized process that encapsulates the role of copper in facilitating cellular proliferation and growth, specifically within the context of cancer biology [161, 162]. This process is indicative of the critical involvement of copper in the promotion of hyperplasia, metaplasia, and potentially neoplastic transformations, serving as a testament to the dualistic nature of metal in oncogenesis [4, 163]. In the realm of cuproplasia, copper acts as a facilitator of cell growth, contributing to the pathological expansion of cells that characterizes tumorigenesis. This copper-dependent proliferation further elucidates the importance of maintaining copper homeostasis within the cellular environment to prevent oncogenic transformation and progression.

The relationship between cuproplasia and cuproptosis is emblematic of the complex interplay between role of copper in both supporting life and inducing death. While cuproplasia elucidates the role of copper in promoting cell growth and proliferation, cuproptosis reveals a therapeutic window where the cytotoxic effects of copper can be harnessed to target and eliminate cancer cells. This dichotomy underscores the critical importance of copper regulation in cancer biology, where an intricate balance between promoting cellular proliferation (cuproplasia) and inducing cell death (cuproptosis) must be carefully managed. Therapeutic strategies aimed at exploiting this balance offer a promising avenue for cancer treatment, leveraging the nuanced understanding of the dual roles of copper to develop interventions that inhibit tumor growth while sparing healthy tissues. The exploration of cuproplasia and cuproptosis thus provides a foundation for future research into copper-mediated pathways in oncology, offering insights into novel therapeutic targets and strategies for combating cancer [162, 164, 165].

### Potential therapeutic strategies for targeting cuproptosis in cancer

The elucidation of cuproptosis mechanisms offers a promising avenue for drug discovery, particularly in the development of copper ionophores or cuproptosis-associated agents, potentially useful in future cancer therapies [166]. Compounds like ES and DSF facilitate cell demise by shuttling copper ions into cells and mitochondria, leading to DLAT oligomerization, diminished Fe-S cluster stability, and Npl4 interaction [167]. Certain antimicrobials also act as copper ionophores, thwarting microbial proliferation by elevating intracellular copper concentrations. Agents such as zinc pyrithione, 4-Br-A23187, and Dimethyldithiocarbamate enhance cellular copper levels, exhibiting antimicrobial activity [168, 169]. Likewise, lipophilic copper complexes with bis(thiosemicarbazone) ligands augment copper ion concentrations in cancer and chlamydial host cells [170, 171] Quinoline derivatives, too, are recognized as copper ionophores, with modifications enhancing their efficacy [172].

Particularly, DSF and ES, both undergoing clinical trials, have garnered significant interest. DSF, an FDA-approved alcohol dependence treatment, is well-tolerated at 125–500 mg daily [173, 174]. ES, initially an anti-cancer agent, proved safe despite limited efficacy in past trials [175]. Nanomedicines combining copper ions and ionophores are being intensively researched, targeting tumors more precisely through cuproptosis [176, 177].

The relationship between cuproptosis induced by copper ionophores and mitochondrial metabolism is crucial in cancer drug development. Tumors with elevated mitochondrial metabolism, like melanoma, breast cancer, leukemia, and certain cancer stem cell-like cells in glioblastoma and cholangiocarcinoma, might be more susceptible to these therapies [178–186]. Drug-resistant tumors, often with high mitochondrial metabolic states, might benefit from combined treatments targeting ﻿epidermal growth factor receptor (EGFR) or B-cell lymphoma 2 (BCL-2) [187, 188]. Copper ionophores could synergize with drugs inducing a high mitochondrial metabolic state in tumors, offering a potential therapeutic approach.

Clinical trials have yet to demonstrate significant efficacy differences between experimental and control groups for drugs like ES. In the phase III clinical trial evaluating ES, no significant difference in treatment effectiveness was observed between the experimental and control groups [175]. However, the impact of ES varied in patients with lower serum lactate dehydrogenase (LDH) levels, suggesting that serum LDH could serve as a biomarker for assessing the potential response to cuproptosis-inducing drugs. This finding indicates the possibility of using serum LDH levels in future clinical settings to determine the appropriateness and potential effectiveness of treatments involving cuproptosis-related drugs.

Furthermore, ongoing research is concentrating on the discovery of new copper ionophores as potential cancer-targeting drugs [189]. These ionophores, with their diverse physicochemical properties, are suitable for treating various types of cancers. Copper complexes, known to elevate intracellular copper ion concentrations and consequently induce cancer cell death, also hold promise as future agents in cuproptosis-based therapies e [190]. The development of copper ionophores for clinical use is particularly notable for the sensitivity of their properties and functions to structural modifications. This is exemplified by the variations in the derivatives of bis(N4-methylthiosemicarbazone) and quinolines, where minor structural changes can significantly alter their therapeutic properties [191–194].

Copper ionophores can be strategically combined with targeted therapeutic agents such as tyrosine kinase inhibitors (TKIs) and proteasome inhibitors (PIs), particularly in cancers characterized by high mitochondrial metabolic activity. LDH levels could be employed both as a predictive marker to guide the initial decision to use these treatments and as a prognostic indicator for monitoring the treatment efficacy. Summarily, the potential of cuproptosis-related therapy in specific cancer patient populations necessitates further investigation to fully understand its viability and to optimize treatment strategies based on individual patient characteristics and tumor profiles.

#### Small compounds inducing cuproptosis in cancer

Cuproptosis, a distinctive cell death mechanism, has generated significant interest in cancer research due to its potential in novel therapeutic approaches, including inhibiting tumor cell proliferation and possibly reversing cancer drug resistance, akin to ferroptosis [195–198]. Emerging compounds have shown the ability to induce cuproptosis in preclinical models. Specifically, the downregulation of FDX1, DLAT, SDHB (succinate dehydrogenase complex, subunit B), and DLST (dihydrolipoamide S-succinyltransferase) genes in primary CRC tissues suggests a link to disease progression, with higher expression of these genes correlating to better prognosis [195–198]. ES-Cu treatment notably reduces cell viability in CRC cells. TTM, a copper chelator, significantly hinders cuproptosis, acting as an inhibitor. Conversely, the glucose metabolism inhibitor 2-deoxy-D-glucose sensitizes cancer cells to cuproptosis [196]. Similarly, galactose and Octyl itaconate (4-OI) promote cuproptosis, with 4-OI enhancing sensitivity through inhibition of GAPDH (glyceraldehyde-3-phosphate dehydrogenase)-mediated aerobic glycolysis[196]. The silencing of FDX1 reverses the cuproptosis-inducing ability of 4-OI. In vivo, 4-OI amplifies the anti-tumor effects of ES-Cu and targets GAPDH to promote cuproptosis [199, 200].

Anisomycin, a p38 MAPK signaling pathway agonist, significantly reduces ovarian cancer stem cell proliferation in vitro, hinting at its potential in cuproptosis induction [199, 200]. It also notably decreases the transcriptional levels of metallothionein, pyruvate dehydrogenase (PDH) complex, lipoid acid pathway, and Fe-S cluster proteins. Impact of Curcumin on ferroptosis and cuproptosis varies among different hepatocellular carcinoma cells, with single-cell transcriptome data suggesting it as a potential cuproptosis inducer [201]. The cuproptosis-related gene Cyclin-dependent kinase inhibitor 2A (CDKN2A) is linked to the malignancy of head and neck squamous cell carcinoma (HNSCC), with plicamycin showing strong binding to CDKN2A and inhibiting HNSCC progression, indicating its role as a cuproptosis inducer [202].

Sorafenib, a multi-tyrosine kinase inhibitor for unresectable hepatocellular carcinoma, functions as a ferroptosis inducer by inhibiting cystine-glutamate transport and depleting GSH [203, 204]. Erastin also induces ferroptosis through similar mechanisms [205, 206]. Recent studies reveal that both sorafenib and erastin enhance the cuproptotic effects of copper ionophore ES and ES-Cu in hepatocellular carcinoma cells by promoting copper-dependent lipoylated protein aggregation, suppressing mitochondrial matrix-related proteases mediated FDX1 protein degradation, and reducing intracellular GSH synthesis [195–198]. This suggests a possible interplay between cuproptosis and ferroptosis, proposing a novel therapeutic strategy for hepatocellular carcinoma that co-targets both processes. These findings reinforce the idea that ferroptosis inducers may also function as cuproptosis inducers, leading to the speculation that agents inducing or inhibiting ferroptosis could similarly affect cuproptosis.

#### Nanoparticles inducing cuproptosis in cancer

﻿In the burgeoning field of oncological therapeutics, nanoparticles (NPs) have emerged as a pivotal tool in the induction of cuproptosis by virtue of their unique physicochemical properties, facilitate the targeted delivery and sustained release of copper ions within the TME. This process is instrumental in initiating cuproptosis. Nanoparticles offer a distinct advantage over traditional delivery systems by overcoming physiological barriers and enhancing the intracellular accumulation of copper ions. This is particularly crucial given the role of copper transport proteins, such as ATP7A. ATP7A is crucial in maintaining exceptionally low intracellular copper levels, posing a challenge to achieve the high cytoplasmic copper concentrations necessary for inducing cuproptosis [161]. However, nanodrug delivery systems have been developed to overcome these barriers. Certain nanoparticles (NPs) show promise as anticancer agents by inducing cuproptosis. For instance, CuMoO4 nanodots may trigger cuproptosis in tumor cells, offering a promising approach for combined cancer therapy [150, 207]. The HD/BER/GOx/Cu hydrogel system reduces dosing frequency and minimizes invasiveness in local cancer treatment, shrinking breast cancer size before surgery and suppressing tumor growth [208].

In this system, released DSF chelates with Cu(II) in situ to form highly cytotoxic bis(diethyldithiocarbamate) copper (CuET), inducing cell apoptosis and cuproptosis [209]. Au@MSN-Cu/PEG/DSF decreases the expression of DLAT, LIAS (Lipoic acid synthase), NPL4 and, in combination with photothermal therapy, effectively kills tumor cells and inhibits tumor growth [152, 209]. NP@ESCu, through intracellular ROS-mediated release of ES and Cu, induces cuproptosis and immune responses, leading to cancer cell death [152]. NP@ESCu has been effective in vitro and in a mouse model with subcutaneous bladder cancer, enhancing the anti-tumor activity of anti-programmed death-ligand 1 (αPD-L1) [152].

TP-M-Cu-MOF/siATP7a targets copper trafficking and enhances copper intake, resulting in cuproptosis and improved therapeutic efficacy in small-cell lung cancer brain metastasis in mice [210]. GOx@[Cu(tz)] increases cancer cell sensitivity to cuproptosis by depleting GSH and glucose. It inhibits tumor growth in athymic mice with 5637 bladder tumors, showing minimal systemic toxicity. Complexes 4 and 6 successfully induce cuproptosis in A2780 cells [7, 211].

The ﻿Platelet vesicle (PV)- coated cuprous oxide (Cu2O) nanoparticles/ Thioredoxin Binding Protein-2 (TBP- 2) cuproptosis sensitization system (PTC) for maintains long-term circulation and tumor inhibiting capacity [212, 213]. In acidic conditions, PTC rapidly degrades, releasing copper ions and hydrogen peroxides in tumor cells. TBP-2 penetrates the cell membrane under light irradiation, producing hydroxyl radicals, depleting GSH, and inhibiting copper efflux. PTC significantly inhibits lung metastasis by inducing cuproptosis in vitro and in vivo, increases central memory T cells in peripheral blood, and prevents tumor rechallenge [213].

CuET, with its lower reduction potential and inertness to GSH, reverses cisplatin resistance in non-small cell lung cancer by inducing cuproptosis [214, 215]. Intracellular GSH levels influence the anti-cancer effect of CuET in overcoming drug resistance in A549/DDP cells. CuET NPs induce cuproptosis in A549/DDP cells, distinct from the apoptosis caused by cisplatin, demonstrating potent antitumor activity and superior biosafety in a cisplatin-resistant tumor model [214, 215].

#### Combination therapies with ICB

Like other cell death mechanisms, such as ferroptosis and pyroptosis, cuproptosis has been identified as a contributor to tumor immunogenicity, potentially augmenting the efficacy of immune checkpoint blockade (ICB) therapies [216–218]. ICB, a forefront in cancer immunotherapy, enhances and activates immune responses, offering a significant alternative to conventional treatments like chemotherapy, radiotherapy, and targeted therapy due to its substantial clinical effectiveness and distinct benefits [219–221]. Prior to the recognition of cuproptosis, it was shown that copper levels in tumors influence PD-L1 expression in cancer cells, suggesting a possible synergistic effect between cuproptosis and ICB therapies [222].

Intratumoral copper levels notably influence PD-L1 expression in tumor cells, evidencing a robust correlation between CTR1 and PD-L1 in numerous cancer types, unlike in corresponding healthy tissue [222]. Enhancing copper levels within tumor cells raises PD-L1 expression, thereby facilitating immune evasion. In contrast, copper chelators such as Dextran-catechin or ﻿Tetraethylenepentamine (TEPA) inhibit phosphorylation of Signal transducer and activator of transcription 3 (STAT3) and EGFR, leading to PD-L1 degradation through ubiquitination and bolstering CD8 + T cell and natural killer cell infiltration to curb tumor growth. Although disulfiram combined with copper does not reduce tumor size, it increases PD-L1 expression and blocks T-cell entry by inhibiting PARP1 activity and enhancing Glycogen synthase kinase 3 phosphorylation at Ser9 via the (poly (ADP-ribose) polymerase family, member 1) PARP1 gene [223]. The combination of anti-PD-1 antibodies with disulfiram/copper further decelerates tumor growth. Copper ions in tumor tissues can be targeted by copper chelators, showcasing their anti-cancer capabilities. A copper-induced hydrogel incorporating anti-PD-L1 and nitric oxide intensifies immunotherapy by promoting immunogenic cell death and curbing cancer-associated fibroblasts, thus improving immune cell infiltration [224].

**Fig. 8:**
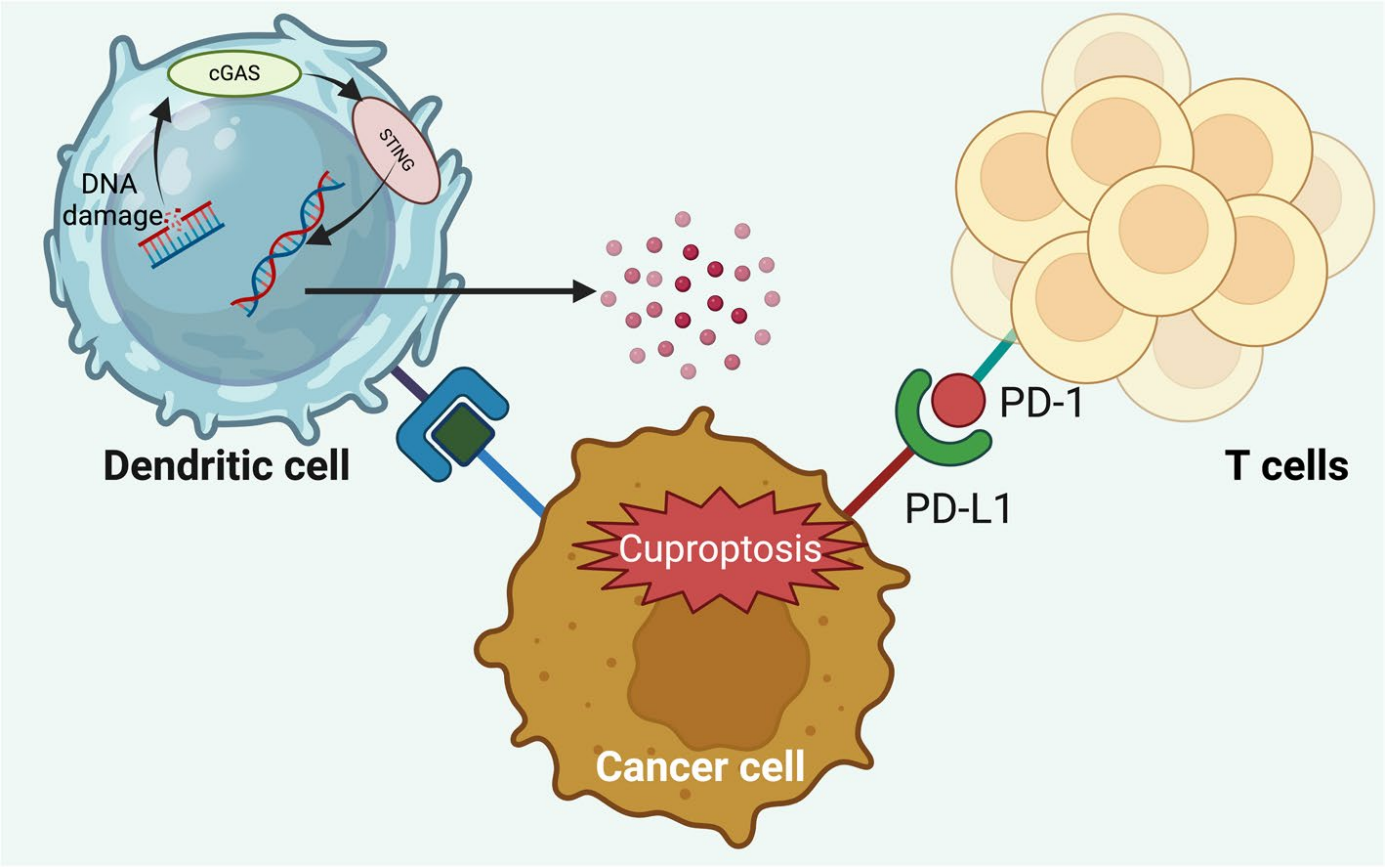
Cuproptosis enhances anti-cancer immune effects by influencing the cGAS-STING pathway. This signaling pathway is activated inside dendritic cells by cancer cells undergoing cuproptosis, triggered by treatments such as ES and copper chloride (CuCl2). This reaction leads to the secretion of pro-inflammatory cytokines and chemokines including IL-2, TNF-α (tumor necrosis factor-alpha), IFN-γ (interferon-gamma), C-X-C motif chemokine ligand (CXCL) 10, and CXCL11. Furthermore, the concurrent use of cuproptosis inducers with PD-1 blockade significantly increases the population of circulating CD45 + CD8 + T cells, thereby enhancing the overall effectiveness of cancer therapy

Copper uptake is facilitated by CD44, a cell surface glycoprotein [225]. Elevated CD44 levels increase mitochondrial copper during macrophage activation, where mitochondrial copper drives metabolic shifts and epigenetic changes causing inflammation through Nicotinamide adenine dinucleotide (NADH) redox cycling. Disulfiram/copper boosts anti-tumor immunity by inducing immune cell death, polarizing M1-macrophages, and altering glucose metabolism via the mTOR pathway [226]. The synergy of disulfiram/copper with CD47 blockade amplifies CD8 + T cell cytotoxicity by aiding dendritic cell maturation [227]. Furthermore, in clear cell renal carcinoma, cuproptosis augments cancer immunity by activating the cGAS-STING (cyclic Guanosine monophosphate—Adenosine monophosphate synthase- stimulator of interferon gene) signaling pathway [228] (Fig. 8).

*Guo *et al*.* engineered NP@ESCu to markedly elevate PD-L1 expression in cancer cells, thereby boosting the impact of immunotherapy [152]. This nanomedicine synergizes the effects of ES and copper in the tumor, prompting cuproptosis and stimulating anti-tumor immune responses, thus improving the performance of anti-PDL-1 antibody therapy [152]. Additionally, *Huang* and team's Buthionine Sulfoximine- Catalase@ Metal–Organic Framework@ n-Dodecyl β-D-maltoside (BSO-CAT@MOF-199@DDM) induces cuproptosis and immunogenic cell death (ICD) [229]. This leads to the release of immunostimulatory substances like ATP, high-mobility group box 1, and calreticulin in cells, reversing the immune-suppressive TME in glioblastoma and increasing cytotoxic T-cell infiltration in the tumor milieu [229]. The concurrent application of BSO-CAT@MOF-199@DDM with αPD-L1 treatment significantly bolsters the anti-tumor effect [229]. Despite these encouraging outcomes in combining cuproptosis with ICB, the exact mechanism by which cuproptosis amplifies tumor immunogenicity is still not fully understood, necessitating further investigation.

#### Combination therapies with ROS-Based therapy

ROS encompass a broad spectrum of oxygen-derived oxidants [230]. While not directly linked to the cuproptosis mechanism, ROS are integral to other copper-induced cell death processes and should not be disregarded [231]. Various therapeutic methods such as radiotherapy [231, 232], photodynamic therapy [233], chemodynamic therapy [234], and sonodynamic therapy [235] have been shown to effectively eradicate tumor cells by generating an excess of ROS. These treatments are promising for their potential to synergize with cuproptosis in targeting tumor cells.

In a system sensitized to cuproptosis, Thioredoxin-binding protein-2 (TBP-2, a type-I aggregation-induced emission photosensitizer) has demonstrated the ability to produce a large number of hydroxyl radicals when exposed to light [236]. This reaction leads to the depletion of intracellular GSH and the accumulation of copper, thereby enhancing the efficacy of cuproptosis [236]. Such an approach has shown substantial success in inhibiting the pulmonary metastasis of breast cancer.

Moreover, ROS are intricately linked to other programmed cell death types like ferroptosis and pyroptosis [237, 238]. Consequently, the strategic combination of therapies that induce various forms of cell death related to ROS can be advantageous in cancer treatment. Building on this concept, a variety of cell death mechanisms, including apoptosis [211, 234], pyroptosis [239], ferroptosis [211], and autophagy [240], have been employed to augment the effects of cuproptosis.

### Translational potential and clinical trials

Over the past years, a substantial amount of research has indicated that copper complexes might be an effective therapeutic approach in cancer treatment [26, 241]. This is based on the ability of copper to induce cell death in cancerous cells through mechanisms such as apoptosis and the build-up of free radicals. These insights into the cuproptosis open new avenues for cancer therapy. A notable development in this field is the creation of a nonporous copper-coordination nanomaterial by *Xu* and colleagues, engineered with glucose oxidase (GOx), known as GOx@[Cu(tz)] [7]. This nanomaterial has demonstrated significant tumor growth inhibition in live models, specifically athymic mice with bladder tumors, achieving a reduction of up to 92% without substantial systemic toxicity [7].

**Fig. 9:**
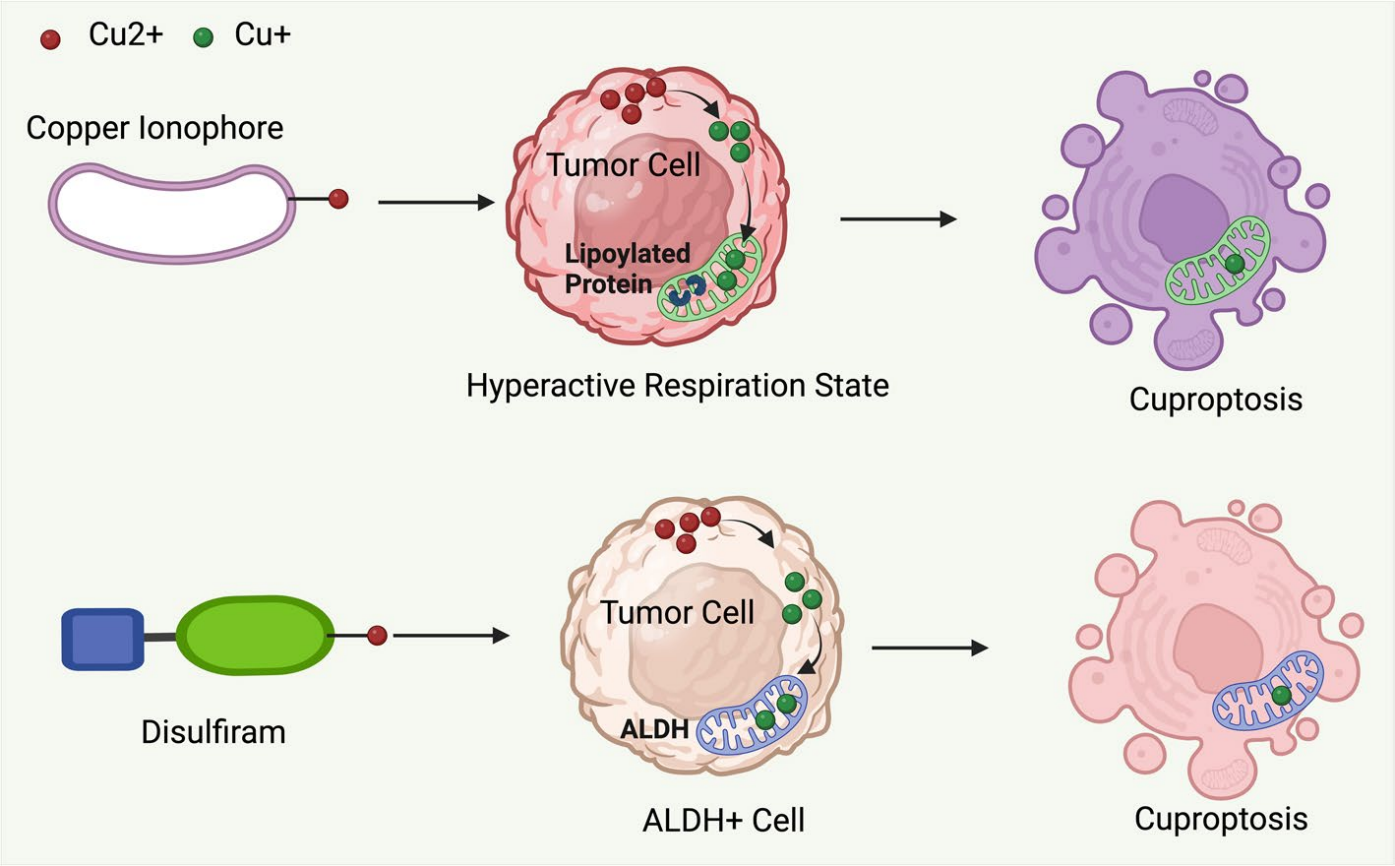
Two potential therapeutic approaches aim to harness cuproptosis for cancer treatment. Firstly, the copper ionophore ES is posited to trigger cuproptosis in cancer cells characterized by elevated expression of lipoylated mitochondrial enzymes or those in an enhanced respiratory state. Secondly, the combination of DSF and copper specifically targets cancer cells exhibiting high levels of ALDH expression, presenting a targeted strategy against cancer cells with this specific metabolic profile

Furthermore, copper ionophores like ES have displayed anticancer properties by prompting the production of ROS in cancer cells [113]. ES seems to show enhanced efficacy in cancer cells that have abundant lipoylated mitochondrial proteins and an increased respiratory state. This was substantiated by a phase III clinical trial that revealed a more pronounced antitumor effect of ES in melanoma patients who had low plasma levels of lactate dehydrogenase, a marker of mitochondrial metabolic activity [175]. In addition, DSF has been found to induce cuproptosis and exhibit antitumor activity against various cancer types, especially when paired with copper ions [1, 242–244]. DSF is particularly effective against cancer stem cells high in aldehyde dehydrogenase (ALDH), potentially reducing the chance of tumor relapse [245–247] (Fig. 9).

However, DSF/Cu have not shown potential in patients with glioblastoma in clinical setting. This study registered with ClinicaTrials.gov (NCT03034135) across multiple institutions demonstrated that DSF/Cu treatment exhibits minimal efficacy in treating IDH-wild type glioblastoma patients without selection and seemingly fails to enhance sensitivity to temozolomide significantly [248]. Another clinical trial (NCT02678975) also indicated that combining disulfiram and copper with standard chemotherapy for the treatment of recurrent glioblastoma did not lead to an increase in patient survival rates [249]. Moreover, a daily treatment with a 400 mg dose of disulfiram was associated with a higher incidence of adverse effects. Consequently, the study concluded that this combination therapy does not confer an advantage for patients with recurrent glioblastoma [249].

These findings collectively suggest further studies are essential in related to the effectiveness of DSF coupled with copper strategies in treating different tyeps of cancer.

### Future perspective

﻿As a recently identified cellular demise mechanism, Cuproptosis remains largely unexplored with only the lipoic acid pathway identified as a crucial mediator.The discovery of the copper death concept relied on the study of copper ionophores that have antitumor effects. The potential involvement of additional metabolic pathways and how lipoylated mitochondrial enzyme aggregation activates copper-dependent signaling leading to apoptosis necessitate further research. This will help clarify the role of cuproptosis across various biological levels and its clinical application potential. Understanding these mechanisms could unveil new therapeutic opportunities, especially in diseases characterized by copper accumulation.

Given the association of copper accumulation with several diseases, including ﻿Wilson’s disease, neurodegenerative conditions, and cancer, it is plausible that cuproptosis could contribute to these conditions, presenting a novel therapeutic avenue. The effectiveness of copper chelators in mitigating copper-induced cell death underscores the importance of copper-deprivation strategies in managing elevated intracellular copper levels. This necessitates further research to elucidate the initiation of cuproptosis and its role in disease progression, highlighting the need for more insights into its dynamic processes.

The recent discovery of cuproptosis lacks specific biomarkers, hindering the assessment of its role in human diseases. This gap significantly constrains the advancement of therapies targeting cuproptosis. Identifying precise and sensitive biomarkers in various diseases is crucial for developing targeted clinical applications and enhancing our understanding of the impact of cuproptosis on pathological conditions.

Another noteworthy point is that while the efficacy of disulfiram in targeting the proteasomal degradation pathway and inducing cell death in cancer cells is noted, it has shown a range of adverse effects [250, 251]. These include but are not limited to hepatotoxicity, neurotoxic symptoms such as encephalopathy and peripheral neuropathy, and psychiatric manifestations such as depression and psychosis [252, 253]. Such adverse reactions can significantly impact patient quality of life and may limit clinical utility of disulfiram.

Furthermore, disulfiram-copper complex can lead to increased oxidative stress, further exacerbating potential damage to normal cells [254, 255]. The reactive nature of the complex may also induce off-target effects, thereby contributing to systemic toxicity [253, 256]. It is crucial for ongoing and future trials to monitor these effects closely, optimize dosing regimens, and develop strategies to mitigate these responses. The incorporation of biomarkers indicative of oxidative stress or organ function may aid in the early detection and management of adverse events. The addition of these insights into the limitations of disulfiram use in cancer therapy can guide future research and clinical strategies to improve therapeutic indices and patient outcomes.

Additionally, the differential impact of cuproptosis on various tissue types underscores the necessity for a nuanced understanding of this cell death pathway. In cancerous tissues, characterized by aberrant metabolic processes and altered copper ion transport mechanisms, there is an enhanced sensitivity to cuproptosis. This suggests that leveraging cuproptosis-inducing agents could be particularly effective in targeting and eliminating cancer cells, sparing healthy cells due to their robust copper regulation capabilities. However, certain normal tissues, such as the liver which plays a central role in copper metabolism, may be more susceptible to the deleterious effects of excess copper, leading to potential tissue damage and dysfunction. Therefore, while developing cuproptosis-based therapeutic strategies, it is crucial to consider the varying responses of different tissue types to minimize potential adverse effects on sensitive organs. This highlights the need for tissue-specific studies in the advancement of cuproptosis as a targeted cancer therapy, ensuring the therapeutic efficacy is maximized while minimizing off-target effects. While cuproptosis holds promise as a targeted cancer therapy, its effects are not uniform across all tissue types [165]. This discrepancy highlights the potential for cuproptosis-inducing agents to selectively target cancer cells without harming normal tissues, emphasizing the importance of tissue-specific studies in the development of cuproptosis-based therapies. To address these complex dynamics, future research should focus on the following objectives: i. Quantitative analysis of FDX1 and DLAT expression levels in a broad spectrum of cancer types, correlating these levels with responsiveness to cuproptosis-inducing agents; ii. Development of robust biomarkers based on the expression profiles of cuproptosis-related proteins to predict therapeutic outcomes; iii. Comparative studies investigating the effects of cuproptosis in cancerous versus normal tissues to ascertain therapeutic selectivity and minimize off-target effects.

High-throughput screenings and AI methodologies are poised to fast-track the discovery of novel agents targeting cuproptosis, enhancing treatment options. Ensuring these compounds are safely and effectively delivered to specific organs will be crucial. Addressing these challenges will deepen our understanding of the role of cuproptosis in disease mechanisms and support the development of precise therapeutic strategies against copper-related conditions. We will further refine our understanding of cuproptosis as a novel therapeutic avenue, offering the potential for highly selective and efficacious cancer treatments.

## Conclusion

In conclusion, the potential of cuproptosis-related therapies in the realm of cancer treatment is undeniable, yet our comprehension of this phenomenon remains cursory at best. The imperative to delve into the intricacies of cuproptosis mechanisms is paramount. Through a meticulous analysis of existing literature and the identification of research gaps, we stand on the cusp of pioneering novel therapeutic modalities. These modalities aim not only to target but also to leverage the unique metabolic reliance of cancer cells on copper. This pursuit necessitates a multidisciplinary collaboration to break free from the confines of current research paradigms and to embrace cutting-edge therapeutic strategies. However, to actualize the promise of these therapies, a more profound investigation into how dysregulated copper levels can guide targeted therapy is essential. Unraveling the intricate ways through which copper influences tumorigenesis, invasiveness, and chemoresistance is crucial for paving the path to groundbreaking treatments. Despite the promise shown in preclinical studies in enhancing the efficacy of immunotherapy, the transition to clinical applications has highlighted concerns regarding specificity and potential collateral damage to non-cancerous cells. The scarcity of specific biomarkers for cuproptosis and the side effects associated with copper-modulating agents represent significant hurdles. The link between altered copper levels and cancer progression is clear. Moving forward, efforts must concentrate on identifying the precise regulatory mechanisms of cuproptosis and developing targeted, safe therapeutic interventions. Only through such focused and collaborative endeavors can we effectively harness the power of cuproptosis for cancer treatment, marking a significant stride towards the innovation of cancer therapeutics.

## Data Availability

No datasets were generated or analysed during the current study.
